# A novel compound heterozygous mutation in *VARS2* in a newborn with mitochondrial cardiomyopathy: a case report of a Chinese family

**DOI:** 10.1186/s12881-018-0689-3

**Published:** 2018-11-20

**Authors:** Keze Ma, Mingyu Xie, Xiaoguang He, Guojun Liu, Xiaomei Lu, Qi Peng, Baimao Zhong, Ning Li

**Affiliations:** 1 Department of Neonatal Intensive Care Unit, Dongguan Children’s Hospital, Dongguan, 523325 Guangdong China; 2 Department of Medical and Molecular Genetics, Dongguan Institute of Pediatrics, Dongguan, 523325 Guangdong China

**Keywords:** Mitochondrial disorders, Cardiomyopathy, *VARS2*, Neonate

## Abstract

**Background:**

Genetic defects in the mitochondrial aminoacyl-tRNA synthetase are important causes of mitochondrial disorders. *VARS2* is one of the genes encoding aminoacyl-tRNA synthetases. Recently, an increasing number of pathogenic variants of *VARS2* have been reported.

**Case presentation:**

We report the novel compound heterozygous pathogenic *VARS2* mutations c.643 C > T (p. His215Tyr) and c.1354 A > G (p. Met452Val) in a female infant who presented with poor sucking at birth, poor activity, hyporeflexia, hypertonia, persistent pulmonary hypertension of newborn (PPHN), metabolic acidosis, severe lactic acidosis, expansion and hypertrophic cardiomyopathy. These heterozygous mutations were carried individually by the proband’s parents and elder sister; the two mutations segregated in the family and were the cause of the disease in the proband.The c.643 C > T (p. His215Tyr) mutation was not described in the ExaC, GNomAD and 1000 Genomes Project databases, and the frequency of c.1354 A > G (p. Met452Val) was < 0.001 in these gene databases.The two mutated amino acids were located in a highly conserved region of the *VARS2* protein that is important for its interaction with the cognate tRNA. The two missense mutations were predicted by online tools to be damaging and deleterious.

**Conclusions:**

Our report expands the spectrum of known pathogenic*VARS2* variants associated with mitochondrial disorders in humans.*VARS2* deficiency may cause a severe neonatal presentation with structural cardiac abnormalities.

## Background

Mitochondrial diseases (MDs) are multisystem disorders that are caused by inherited or acquired mutations in either the mitochondrial DNA or nuclear DNA known to encode mitochondrial proteins [1]. Mitochondrial aminoacyl-tRNA synthetases (mt-aaRSs) are key enzymes in mitochondrial protein synthesis that catalyze the binding of amino acids to their specific tRNAs (http://www.Uniprot.org).

The *VARS2* gene is one of the genes encoding mt-aaRSs. Few individual patients with *VARS2* deficiencies have been described with specific phenotypes. The phenotypes present at any time during life and demonstrate great clinical heterogeneity. Mutations in the *VARS2* gene are associated with encephalomyopathy or cardiomyopathy and result in chronic disability and a poor prognosis [2–4]. These patients can present with structural brain abnormalities, hypotonia, psychomotor delay, seizures, feeding difficulty, severe lactic acidosis, hypertrophic cardiomyopathy, and abnormal cranial magnetic resonance imaging (MRI) [2, 5].

In the present study, we found a novel compound heterozygous variant of the *VARS2* gene [c.643 C > T (p.His215Tyr) plus c.1354 A > G (p.Met452Val)] in the proband by targeted capture and sequencing. p.His215 and p.Met452 were located in the aminoacyl-tRNA synthetase (also known as amino acid translation or aminoacyl-tRNA ligase). The proband was diagnosed with mitochondrial cardiomyopathy and presented with poor sucking, hypertonia, severe lactic acidosis, hypertrophic cardiomyopathy and severe pulmonary hypertension and died on the sixteenth day of life due to cardiac arrest.We present detailed clinical data and further delineate the phenotype associated with this disease.

## Case presentation

**Fig. 1:**
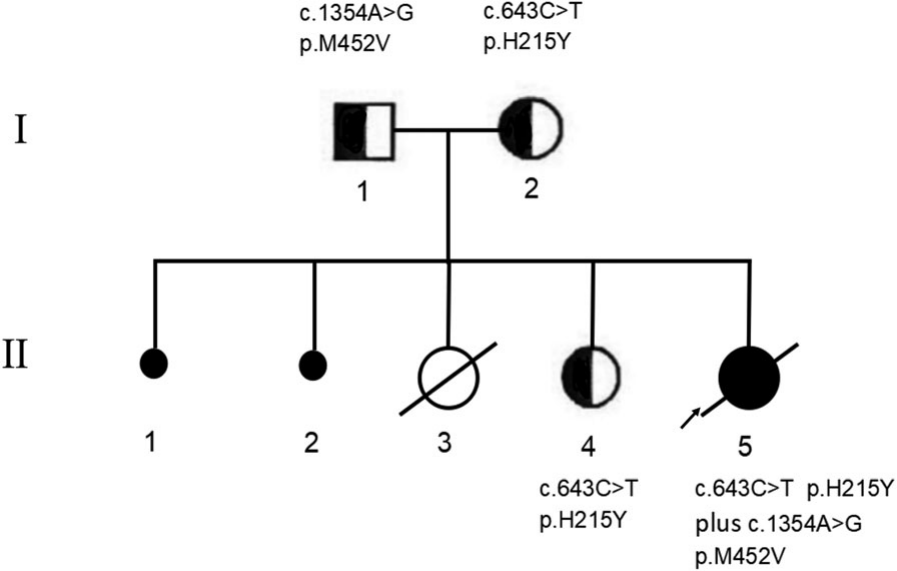
Pedigrees of the family members are shown documenting segregation of the alleles

The female proband was the fifth child of non-consanguineous parents of Han Chinese descentand was born at 38 weeks gestation by Cesarean section delivery due to a uterine scar to a 30-year-old woman following an uneventful pregnancy. The first child of the parents was an unexplained spontaneous abortion, and the second child was an abortion due to a heterotopic pregnancy. The third child died soon after birth with an unknown diagnosis in a grass-roots hospital. The fourth child had a normal phenotype (Fig. 1). The family had no metabolic disorders. The proband had no postnatal adaptation, and the Apgar score was 10 at 1 min. Her birth weight was 2.64 kg (between the 3^rd^ and 10^th^ percentiles). Her head and abdominal circumferences were 32 cm (10^th^ percentile). Her length was 49 cm (50^th^ percentile).

The newborn presented with poor sucking at birth and was transferred to the neonatal intensive care unit due to poor vigor, groaning, shortness of breath and cyanosis, and shock at the sixth day of life. Laboratory analyses found metabolic acidosis and severe lactic acidosis based on the arterial blood gas results, including pH 7.167, pCO_2_ 16.9 mmHg, pO_2_ 50.4 mmHg, HCO_3_–6.2 mmol/L, BE − 22.6 mmol/L, and lactate 13.7 mmol/L (reference ranges: arterial pH 7.35–7.45, pCO_2_ 35–45 mmHg, pO_2_ 60–90 mmHg, HCO_3_–21-24 mmol/L, BE − 3-3 mmol/L, and lactate ≤2.5 mmol/L). Albumin, normal saline and vasoactive agents (dopamine and dobutamine) were used to improve circulation. The acidosis was treated with sodium bicarbonate, but the plasma lactate acid was still 15.6 mmol/L. Coenzyme A and adenosine triphosphate were used to improve the acidosis, but the plasma lactate acid fluctuated between 3.0 and 10.9 mmol/L. She did not present pronounced urinary lactate. Further metabolic work-up revealed an abnormal increase in N-acetyl tyrosine-2 in the urinary organic acid test, but no abnormal acylcarnitine profiles and amino acids were detected.

Echocardiography revealed the presence of right atrial and ventricular expansion, right ventricular hypertrophy, a normal ventricular ejection function, interventricular septum thickening, tricuspid regurgitation and severe pulmonary hypertension at admission. The percutaneous blood saturation revealed 15% variation before and after the catheter. The PPHN was treated with sidenafil. The electrocardiograph showed nodal tachycardia, right ventricular hypertrophy and movement of the ST segment down 0.1 mv at V1 and V2. The Non-Invasive Cardiac System showed tachycardia, a high cardiac output, a reduction in left ventricle systolic function, and high total peripheral resistance at admission. The chest CT scan and three-dimensional reconstruction displayed coarctation of the aorta and right lung pneumonia. The renal and hepatic function tests, creatine kinase, lactate dehydrogenase, ammonia and total homocysteine were normal. The abdominal ultrasound was also normal.

On the physical examination, there were some signs of shock and mild dehydration. The neurological examination revealed poor reactivity, bregma depression with normal size, hyporeflexia and mild hypermyotonia. No seizures, nystagmus, laryngeal stridor or apnea were present in the neurological signs. The amplitude-integrated electroencephalogram (aEEG) indicted a mild abnormality (she showed no obvious sleep-wake cycles). The brain ultrasonic examination revealed mild echo enhancement on the side of the bilateral paraventricular parenchyma, a left-ependymal cyst and a right-choroid plexus cyst. The laboratory investigations showed that she had mild anemia. The child died on the sixteenth day of life due to cardiac arrest.

## Materials and methods

### DNA extraction and sequencing

Genomic DNA from the family was extracted from peripheral whole blood samples using the SolPure Blood DNA kit (Magen) according to the manufacturer’s instructions.The genomic DNA of the three patients was fragmented with the Q800R Sonicator (Qsonica) to generate 300–500-bp fragments.

The paired-end libraries were prepared according to the Illumina library preparation protocol. Custom-designed NimbleGen SeqCap probes (Roche NimbleGen, Madison, WI, USA) were used for in-solution hybridization to enrich the target sequences. The enriched DNA samples were indexed and sequenced on a NextSeq500 sequencer (Illumina, San Diego, CA, USA) with 100,150 cycles of single end reads according to the manufacturer’s protocols.

DNA from the patient’s sister was validated by Sanger sequencing according to parents’ and the proband’s sequencing results.

### Variant annotation and interpretation

The primary data were transformed to the FASTA format after the image analysis, and base calling was conducted using the Illumina Pipeline. The data were filtered to generate ‘clean reads’ by removing adapters and low-quality reads (Q20). The sequencing reads were mapped to the reference human genome version hg19 (2009–02 release, http://genome.ucsc.edu/). Nucleotide changes observed in the aligned reads were called and reviewed using the NextGENe software (SoftGenetics, State College, PA, USA). In addition to the detection of deleterious mutations and novel single nucleotide variants, a coverage-based algorithm developed in-house (eCNVscan) was used to detect large exonic deletions and duplications. The normalized coverage depth of each exon of a test sample was compared with the mean coverage of the same exon in the reference file to detect copy number variants (CNVs).

Sequence variants were annotated using population and literature databases, including 1000 Genomes (http://www.1000genomes.org/), dbSNP (http://www.ncbi.nlm.nih.gov/), GnomAD (http://gnomad.broadinstitute.org/), Clinvar (https://www.st-va.ncbi.nlm.nih.gov/clinvar/), HGMD (http://www.hgmd.cf.ac.uk/ac/index.php) and OMIM (https://www.omim.org/). Online software (https://www.uniprot.org/) was used to analyze the protein structure, predict the conserved and functional domains and perform a multiple sequence alignment. Variant interpretation was performed according to the American College of Medical Genetics (ACMG) guidelines [6]. The possible pathogenicity was predicted according to the online tools MutationTaster, PolyPhen-2, SIFT and MutationAssessor.

### Homology modeling

Homology modeling of the *VARS2* gene in amino acid region 129–1079 was performed by employing the Protein Data Bank (PDB) structure 1gax.1B from UniProt and SwissModel as the template. All side chain atoms in the template structure were deleted. Novel sites and different functional regions were labeled using Chimera 1.12. We show the location of the compound heterozygous mutation on the partial *VARS2* protein structure (Fig. 3b).

## Results

### Sequence analysis

**Fig. 2:**
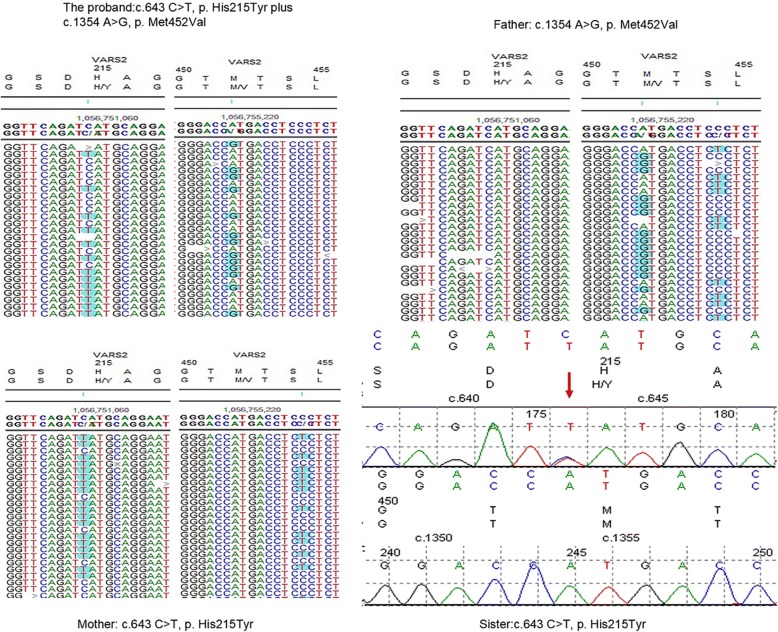
The DNA sequencing results of the proband and her family (including a number of bases around p.H215 and p.M452)

The DNA sequence analysis showed that the proband presented a compound heterozygous missense mutation c.643 C > T (p.His215Tyr) in exon 5 and a missense mutation c.1354 A > G (p.Met452Val) in exon 13 of the *VARS2* gene (NM_001167734.1). The c.643 C > T (p. His215Tyr) mutation was not described in the ExaC, GNomAD and 1000 Genomes Project databases, and the frequency of c.1354 A > G (p. Met452Val) was < 0.001 in these gene databases. Her mother and sister were heterozygous carriers of the c.643 C > T (p.His215Tyr) mutation, and the father was a heterozygous carrier of the c.1354 A > G (p.Met452Val) mutation (Fig. 2).

**Fig. 3:**
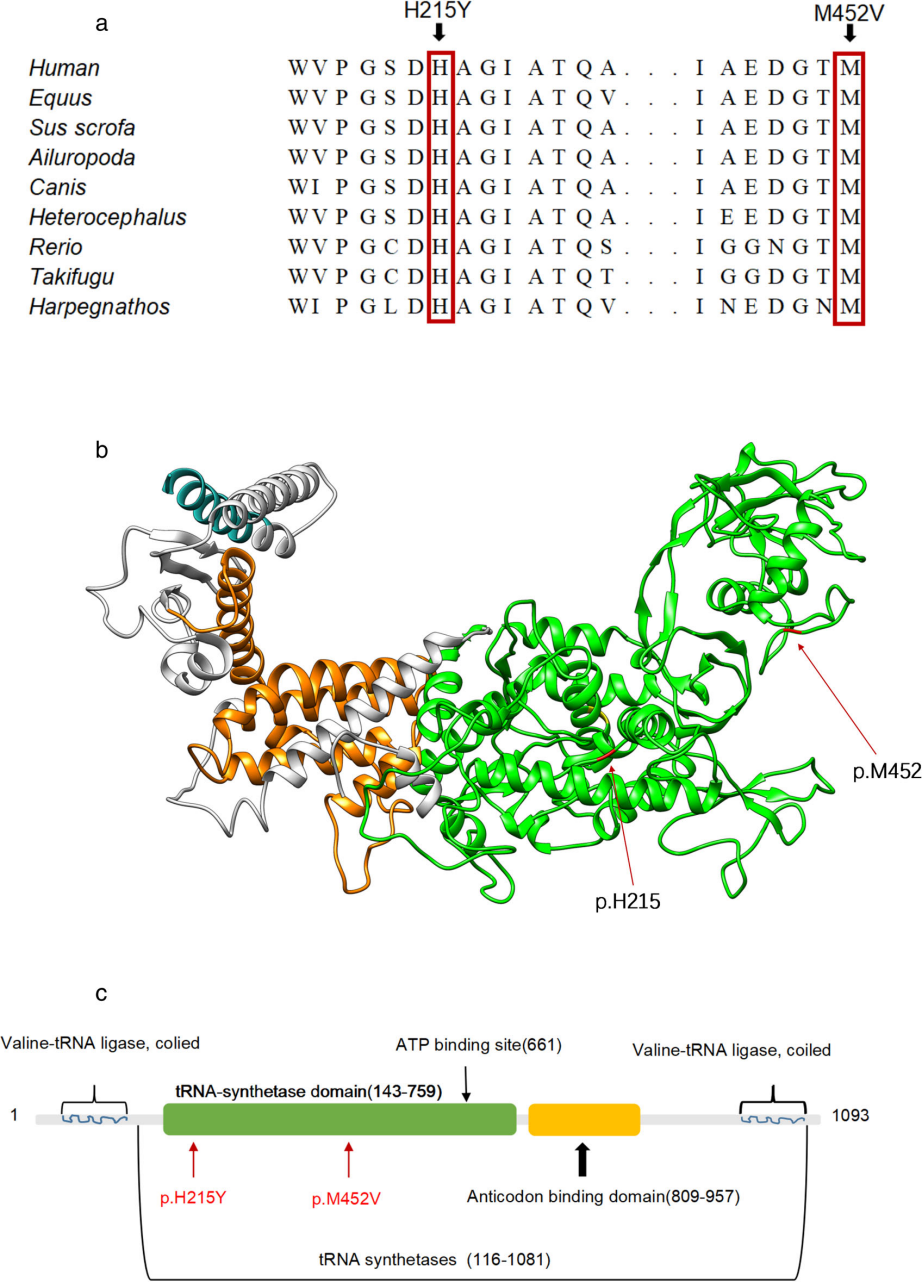
Schematic diagram and molecular model of the *VARS2* gene. **a** VARS2 sequence alignment among representative species around the sites of the missense mutations (p.H215Y and p.M452 V). **b** Homology model of VARS2 (partial amino acids from positions 129 to 1079). Different colors represent different functional regions: the compound heterozygous mutation (p.His215 and p.Met452) is shown in red, the valine-tRNA ligase region in blue, the ATP biding site in yellow, the tRNA-synthetase domain in green and the anticodon binding domain in orange. **c** Schematic representation of the VARS2 protein. The novel compound heterozygous mutation reported here coincides with the tRNA-synthetase domain

The two missense mutations c.643 C > T (p.His215Tyr) and c.1354 A > G (p.Met452Val) are located in regions important for the interaction of the *VARS2* protein with the cognate tRNA. We also analyzed this amino acid region in different species. The result indicated that c.643 C > T (p.His215Tyr) and c.1354 A > G (p.Met452Val) were located in a highly conserved region of the protein (Fig. 3a). The variant *VARS2*: c.643 C > T (p.His215Tyr) was predicted to be “disease causing” by MutationTaster, “probably damaging” by PolyPhen-2 with a score of 1.000 (sensitivity: 0.00; specificity: 1.00), “Affect protein function” by SIFT, and “high” on “function impact” by MutationAssessor. The variant *VARS2*: c.1354 A > G (p.Met452Val) was predicted to be “disease causing” by MutationTaster, “medium” on “function impact” by MutationAssessor, “tolerated” by SIFT, and “benign” by Polyphen-2 with a score of 0.104 (sensitivity: 0.91; specificity: 0.69).However, two amino acid mutations in a highly conserved region of *VARS2* may seriously impact normal protein function in mammals.

## Discussion and conclusions

Variants of the *VARS2* gene, which is located on chromosome 6p21 and encodes a mt-aaRS, are one cause of MDs, although this same gene deficiency may be associated with different clinical phenotypes (Table 1). Recent reports identified variants in this gene associated with mitochondrial encephalopathy, encephalocardiomyopathy and cardiomyopathy [2]. However, how the same gene deficiency can cause these heterogeneous phenotypes remains poorly understood. One hypothesis indicated the gene was associated with the tissue-related amino acid concentrations. Our report further expands the clinical phenotype related to *VARS2* deficiency. Here, we described a newborn with novel compound heterozygous *VARS2* mutations[c.643 C > T (p.His215 Tyr) and c.1354 A > G (p.Met452Val)]who initially presented with poor sucking, hypertonia, respiratory distress, cyanosis, persistent pulmonary hypertension of newborn, right atrial and ventricular hypertrophy, interventricular septum thickening, tricuspid regurgitation, metabolic acidosis and severe lactic acidemia and was diagnosed with neonatal cardiomyopathy.

**Table 1:** Clinical, laboratory, and radiological features associated with VARS2 related mitochondrial disease

Variants	Other clinical signs	Neurological signs	Metabolic screening	Cardiological ultrasound	EEG	Head MRI	Reference
Homozygous c. 1100C > T (p. Thr367lle)	No	Poor sucking, hypotonia, developmental delay, seizures, ataxia, nystagmus, microcephaly, limb spasticity	Lactic acidosis,	No	Epileptic abnormalities	Cerebellar atrophy, T2 hyperintensity of symmetrical periventricular white matter/corpus callosum	[2, 5]
Heterozygous c.1100C > T (p.Thr367lle) plus c.1490G > A (p.Arg497His)	Cryptorchidism, chronic pancreatiti	Stridor, respiratory failure, hypotonia / hypertonia, developmental delay, limb spasticity, poor sucking, poor activity	Lactic acidosis	Hypertrophic cardiomyopathy	Epileptic abnormalities	Cerebral atrophy, hypoplasia of the vermis, T2 hyperintensity of thalamus and septum pellucidum	[2]
Homozygous c. 1258G > A (p.Ala420Thr)	hepatosplenomegaly	Poor sucking, hypotonia, respiratory failure	Severe metabolic acidosis and lactic acidosis	Hypertrophic cardiomyopathy, biventricular dilation	NA	NA	[2]
Heterozygous c.2557-2A > G plus c.1100C > T (p. Thr367lle)	Unusual facial features, congenital hip dislocation,	Hypotonia, hyporeflexia, microcephaly, stridor, irritability, staring episodes	lactic acidosis, urinary lactate, increased plasma alanine	Hypertrophic cardiomyopathy, moderate pericardial effusion,	NA	Cerebellar atrophy, moderate to severe diffuse cerebral	[2]
Heterozygous c.1100C > T (p.Thr367lle) plus c.1150G > A (p.Asp384Asn)	No	Hypotonia, poor sucking, poor activity, developmental delay, seizures	lactic acidosis, increased plasma pyruvate	Hypertrophic cardiomyopathy	Epileptic abnormalities	Cerebellar atrophy, hypoplasia of corpus callosum	[2]
Heterozygous c.1546G > T (p.Glu516*) plus c.2239G > A (p.Ala747Thr)	No	Poor sucking, hypertonia, stridor, developmental delay	lactic acidosis, urinary lactate	Hypertrophic cardiomyopathy and severe pulmonary hypertension	NA	NA	[2]
Heterozygous c.1135G > A (p.Ala397Thr) plus c.1877C > A (p.Ala626Asp)	No	Hypotonia, myasthenia, developmental delay, dyspraxia, seizures	NA	Hypertrophic cardiomyopathy	NA	T2 hyperintensity of the peritrigonal white matter	[2]
Heterozygous c.601C > T (p.Arg201Trp) plus c.1100C > T (p.Thr367Ile)	No	Hypertonia, apnea, microcephaly, exotropia, seizures	Lactic acidosis, urinary lactate and pyruvate, 2-Oxoisocaproic acid and 2-Oxo-3-methyl-N-valeric acid, plasma elevation of alanine	Hypertrophic cardiomyopathy, left ventricular function reducing	Epileptic abnormalities, resembling burst suppression patterns	Hypoplasia of the corpus callosum, the cerebellum accompanied by edema of the brain stem and the frontal white matter	[7]
Heterozygous c.643C > T (p.His215Tyr) plusc.1354A > G (p. Met452Val)	PPHN	Poor sucking, hypertonia, poor activity, hyporeflexia	Severe metabolic acidosis and lactic acidosis	Hypertrophic cardiomyopathy	mild abnormal (showed no obvious sleep-wake cycles)	NA	This study

*NA* not available information

Most recent studies have indicated patients primarily present with a phenotype characterized by severe neonatal encephalocardiomyopathy with hypotonia, poor sucking, respiratory failure, lactic acidosis and abnormal heart anatomy [2, 5, 7]. For example, Fabian B. et al. [5] reported that a newborn with compound heterozygous variants c.601 C > T (p.Arg201Trp) plus c.1100 C > T (p.Thr367Ile) presented with hypertonia, focal seizures, and apnea and developed lactic acidosis. Cardiological ultrasound revealed severe hypertrophic left ventricular dilations and reduced function. The echocardiographic (ECG) examination revealed a severe hypertrophic left ventricle accompanied by moderate dilation and a decrease of the ejection fraction.The brain MRI displayed structural brain abnormalities at the age of five weeks, such as hypoplasia of the corpus callosum and the cerebellum and edema of the brain stem and the frontal white matter. Francesco B et al. [2] described a newborn (c.2557-2A > G plus c.1100 C > T, p.Thr367Ile) who presented with hypotonia, stridor, apnea, hyporeflexia, irritability and intermittent lactic acidosis. The cardiological ultrasound revealed biventricular hypertrophy and pericardial diffusion. The head MRI displayed cerebellar atrophy and a lactate peak at the MR. The described patient herein initially presented with poor sucking, hypertonia, PPHN, severe lactic acidosis and abnormal heart anatomy. However, the patient lacked a head MRI due to the parents’ refusal.

The most frequent variant was homozygous variant c.1100C > T (p.Thr367lle) according to previous reports, including six cases to date [2, 7]. However, their clinical signs were not exactly consistent. Most patients with homozygous variant c.1100C > T (p.Thr367lle) presented with hypertonia, developmental delays, microcephaly, and seizures but no cardiomyopathy [2, 7]. These data could suggest that the homozygous variant c.1100C > T (p.Thr367lle) might have a lesser effect on the heart. Hypertonia, seizures, developmental delays, and lactic acidosis seem to be common signs of *VARS2*-related MD, but seizures and developmental delays usually appear later in life [2, 5, 7]. Our case died at six days of life and did not present epilepsy or developmental delays, which might be due to the improper timing. Additionally, our proband initially presented with hypertonia but not hypotonia, which was similar to previous patient with compound heterozygous variantsc.601 C > T (p.Arg201Trp) plus c.1100C > T (p.Thr367lle) [5] or c.1546 G > T (p.Glu516) plus c.2239G > A (p.Ala367Thr) [2]. PPHN was the notable feature of our proband, which was less often described in previous reports. Only a patient with the compound heterozygous variants c.1546 G > T (p.Glu516) plus c.2239G > A (p.Ala367Thr) was described with severe pulmonary hypertension and death at 19 months [2].

Most patients with variants of *VARS2* have a poor prognosis. However, few patients die during the neonatal period. Francesco et al. [2] reported a case of a premature death with a homozygous mutation c.1258 G > T (p.Ala420Thr) in *VARS2* who presented with hypotonia and poor sucking at birth, followed by respiratory failure, severe metabolic acidosis, lactic acidosis and hypertrophic cardiomyopathy. He died on the ninth day of life. The present report describes a newborn death at sixteen days of life.

p.His215 and p.Met452 are located in the tRNA-synthetase domain (Fig. 3c). Previous studies have indicated that most patients with *VARS2* variants in that region have great clinical impairment or a poor prognosis [2, 5, 7]. Additionally, p.His215 and p.Met452 involve residues that are highly conserved among phylogenetically distant organisms, allowing us to suggest that these variants may affect the 3-dimensional conformation of the *VARS2* protein. However, further study is needed to confirm this hypothesis. This finding highlights the functional importance of the site and suggests that variants may change the structure of the *VARS2* protein and cause defective binding of the tRNA. Based on the ACMG criteria [6], there is some evidence for pathogenicity of the novel compound heterozygous mutations, including two moderate (PM1 and PM2) and four supporting (PP1 to PP4) pieces of evidence. Therefore, the two novel mutations in *VARS2* may be classified as likely pathogenic variants. In future studies, we will focus on developing transgenic animal models.

The discovery of novel variants further expands the spectrum of known *VARS2* mutations in humans.*VARS2* deficiency may cause a severe neonatal presentation with structural cardiac abnormalities. In future studies, we will focus on developing transgenic animal models carrying the variants to characterize the mechanism by which *VARS2* deficiency leads to mitochondrial disease.

## Abbreviations

ACMG: American College of Medical Genetics
ATP: Adenosine triphosphate
ECG: Echocardiography
MDs: Mitochondrial disorders
MRC: Mitochondrial respiratory chain
MRI: Magnetic resonance imaging
mt-aaRS: Mitochondrial aminoacyl-tRNA synthetases
mtDNA: Mitochondrial DNA
nDNA: Nuclear DNA
OXPHOS: Oxidative phosphorylation
PDB: Protein data bank
PPHN: Persistent pulmonary hypertension of newborn
